# Stabilization of human telomeric RNA G-quadruplex by the water-compatible optically pure and biologically-active metallohelices

**DOI:** 10.1038/s41598-020-71429-5

**Published:** 2020-09-03

**Authors:** Jaroslav Malina, Peter Scott, Viktor Brabec

**Affiliations:** 1 Institute of Biophysics, Czech Academy of Sciences, Kralovopolska 135, 61265 Brno, Czech Republic; 2 Department of Chemistry, University of Warwick, Gibbet Hill Road, Coventry, CV4 7AL UK

**Keywords:** Biophysics, Chemical biology

## Abstract

RNA G-quadruplexes have been suggested to play key roles in fundamental biological processes and are linked to human diseases. Thus, they also represent good potential therapeutic targets. Here, we describe, using the methods of molecular biophysics, interactions of a series of biologically-active supramolecular cationic metallohelices with human telomeric RNA G-quadruplex. We demonstrate that the investigated metallohelices bind with a high affinity to human telomeric RNA G-quadruplex and that their binding selectivity considerably differs depending on the dimensions and overall shape of the metallohelices. Additionally, the investigated metallohelices inhibit DNA synthesis on the RNA template containing four repeats of the human telomeric sequence by stabilizing the RNA G-quadruplex structure. Collectively, the results of this study suggest that stabilization of RNA sequences capable of G-quadruplex formation by metallohelices investigated in this work might contribute to the mechanism of their biological activity.

## Introduction

Nucleic acids containing G-rich sequences can form a non-canonical tetrahelical structure called a G-quadruplex^1^. In G-quadruplexes, four coplanar guanines are hydrogen-bonded to one another via Hoogsteen base pairs involving a total of eight hydrogen bonds, forming a planar complex (G-quartet). These G-quartets are stabilized by a central counterion, typically K^+^, and stack upon each other forming stable structures^2^.

Besides DNA, guanine-rich regions in RNA can also fold up into quadruplex structures. While DNA G-quadruplexes have been investigated for decades, such structures formed by RNA came into the focus of research considerably later. The main structural characteristics of RNA quadruplexes are similar to those of DNA G-quadruplexes, however there are significant differences. In contrast to DNA G-quadruplexes, which can adopt a number of quadruplex topologies^3^, RNA quadruplexes are believed to adopt only two structure types: a parallel G-quadruplex structure in which the strands all run in the same direction, and a more recently identified intramolecular antiparallel G-quadruplex structure^4^. Moreover, the stability of RNA G-quadruplexes is higher than that of DNA G-quadruplexes^5^.

Thousands of sequences capable of forming RNA G-quadruplexes have been identified within the human transcriptome^6^. There is circumstantial evidence that these structures exist in living cells^7–10^. They are frequently located in cellular regulatory sequences such as promoters, telomeres and the 5′ untranslated regions (5′ UTRs) of many genes, including genes of clinical interest^11–13^. Interestingly, 5′-UTR G-quadruplex structures have been shown to act as translational repressors^6,13,14^.

RNA G-quadruplexes have been suggested to modulate various biological mechanisms such as mRNA splicing, translation, localization, polyadenylation or regulation of miRNA precursor processing^6,15,16^. Additionally, RNA G-quadruplexes have been shown to be involved in the regulation of HIV-1 replication^17^ and G-rich sequences were found in the genom of several other viruses^18–20^.

Therefore, these structures represent good potential therapeutic targets^21,22^. Despite the growing knowledge about the importance of RNA quadruplexes, reports on their interaction with small molecules are relatively scarce^23–25^. For instance, the cationic 5, 10, 15, 20-tetra(*N*-methyl-4-pyridyl) porphyrin TMPyP4, which has been shown to stabilize many DNA G-quadruplexes^26–28^, can destabilize and unfold highly stable RNA G-quadruplexes^29,30^. Additionally, naphthalenediimide derivatives have been shown to strongly stabilize a human telomeric DNA quadruplex, and a telomeric RNA quadruplex (TERRA), with weak stabilization of a duplex DNA^31^.

**Figure 1:**
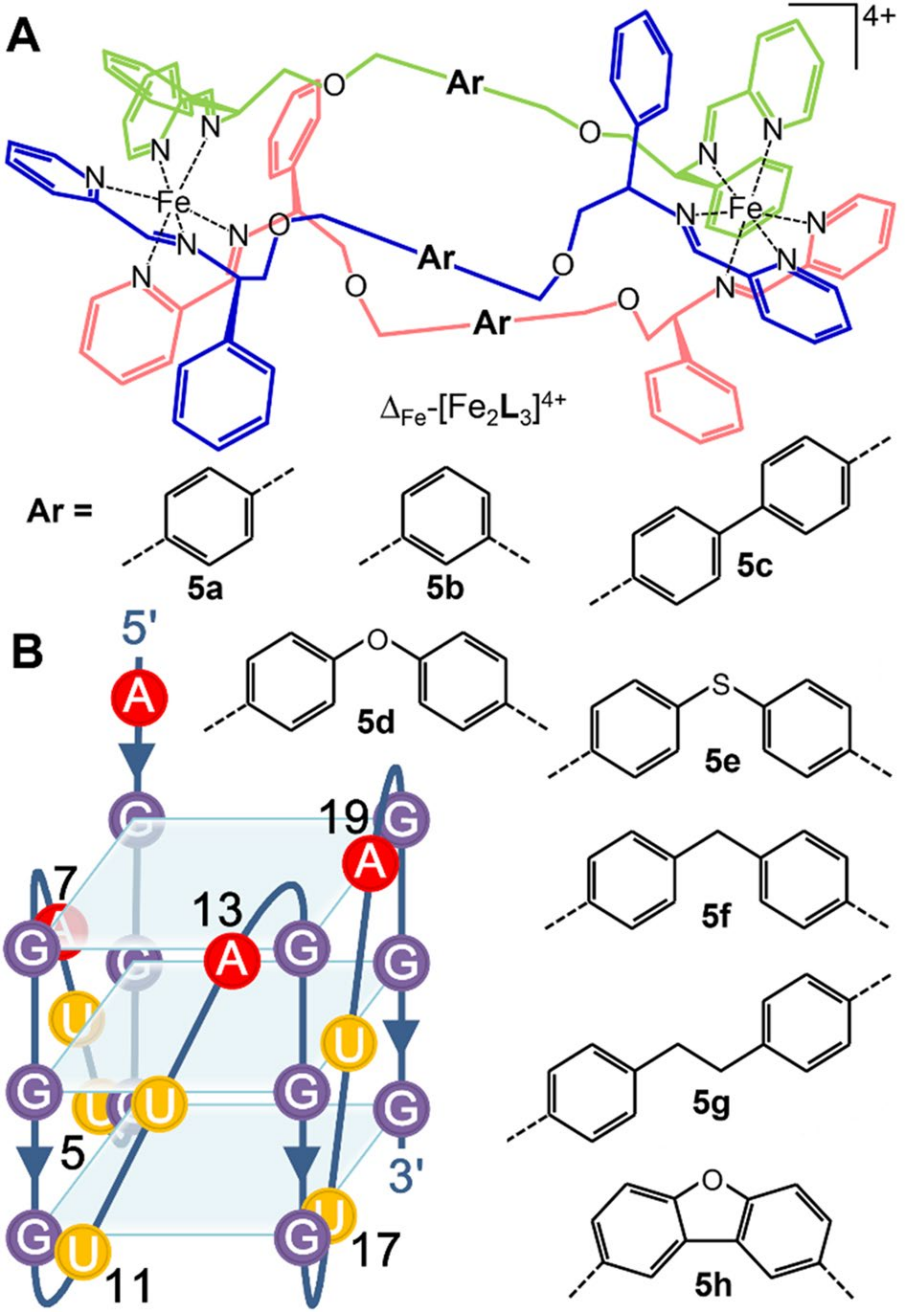
(**A**) Diastereomerically pure Fe(II) metallohelices **5a**–**h**. (**B**) Scheme of the human telomeric RNA (TERRA) G-quadruplex.

It has been shown that chiral Fe(II) and Ni(II) based supramolecular helicates ([M_2_(**I**)_3_]Cl_4_; **I** = C_25_H_20_N_4_), as a type of metal complexes are able to stabilize human telomeric DNA G-quadruplex and inhibit telomerase activity^32, 33^. Another example of a chiral metallohelical complex **5a** (see structure in Fig. 1A) capable of enantioselective stabilization of human telomeric hybrid DNA G-quadruplex and inhibition of telomerase activity was reported recently^34^. This compound developed by Scott and co-workers belongs to the class of cationic metallohelices that are based on helical arrays of fully-encapsulated Fe ions connected by different linking bridges (Fig. 1A)^35^. Unlike above mentioned supramolecular helicates [M_2_(**I**)_3_]Cl_4_, metallohelices are prepared as pure enantiomers and exhibit high kinetic stability in aqueous solutions^35^. Metallohelix **5a** was shown to exhibit promising antimicrobial activity^35^ and served as a prototype compound for the development of a new class of metallohelices **5b**–**h** (Fig. 1A) with structure-dependent activity against Gram-positive and -negative bacteria^36^. Additionally, we showed recently^37^ that for instance, metallohelices **5b** and **5f** accumulate in eukaryotic cells and that a significant fraction of the metallohelices accumulates in the cell nucleus allowing them to interact also with nuclear nucleic acids.

We recently studied the interaction of **5b**–**h** with various DNA G-quadruplexes including human telomeric G-quadruplex and our results revealed that some of the metallohelices prefer binding to G-quadruplexes over double-helical DNA (Ref.^36^ and unpublished data). The results suggest that the high binding affinity of **5b** to DNA G-quadruplexes might contribute to its biological activity.

In this report, we demonstrate by using FRET melting assays, FID assays, gel electrophoresis and other methods that **5a**–**h** are able to recognize and stabilize human telomeric RNA (TERRA) G-quadruplex. Unlike its DNA counterpart, TERRA G-quadruplex folds in potassium^21,38,39^ and sodium^40^ solutions exclusively into a single topology – all strands parallel form (Fig. 1B).

## Results and discussion

### FID assays

**Figure 2:**
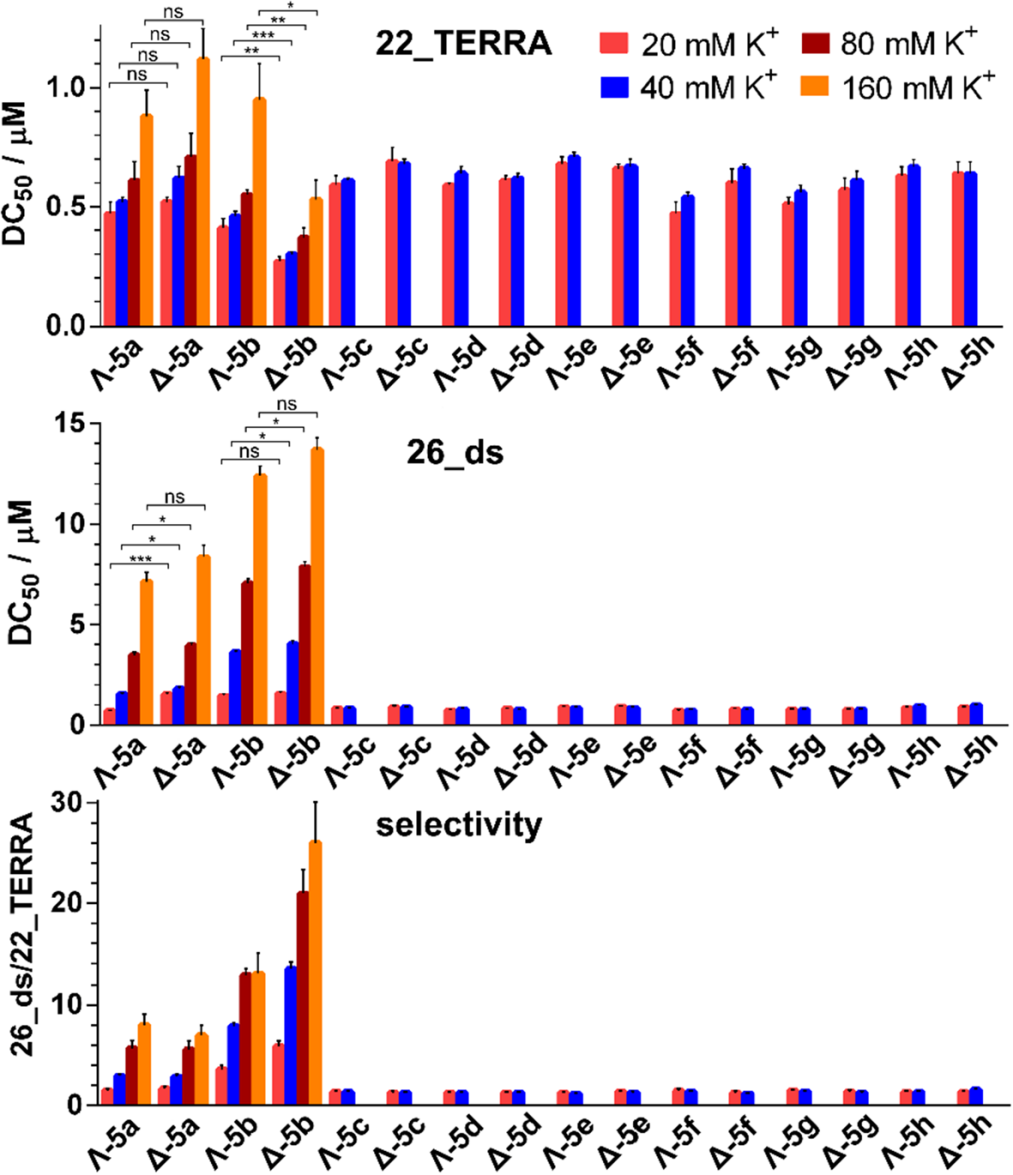
DC_50_ values (μM) for the **22_TERRA** (upper panel) and **26_ds** (middle panel) determined by FID upon addition of the metallohelices in 10 mM potassium phosphate buffer (pH 7) and various concentrations of KCl. Binding selectivity (lower panel) was calculated as a ratio between DC_50_ values for the **26_ds** and the **22_TERRA**. Data shown are expressed as the mean of two independent experiments; error bars indicate the standard error of the mean. Symbols (*, **, ***) and (ns) at the top of the bars indicate statistically significant difference (p ≤ 0.1, p ≤ 0.05, p ≤ 0.01) and statistically insignificant difference (p > 0.1), respectively.

The binding affinities of **5a**–**h** towards TERRA G-quadruplex were evaluated by using the fluorescent intercalator displacement (FID) assay. This widely used method to compare affinities of various ligands to different G-quadruplexes is based on the displacement of thiazole orange (TO)^41^. The RNA G-quadruplex (**22_TERRA**) and the duplex (**26_ds**) (for its sequence see “Experimental section”) were treated with TO, yielding a fluorescence increase upon binding. The addition of metallohelices resulted in a decrease in the fluorescence intensity due to the displacement of bound TO (see Supplementary Figure S1), where the percent fluorescence reduction is directly related to the extent of binding. The concentrations of **5a**–**h** required to give a 50% decrease in TO fluorescence (DC_50_ values) were determined and the results are shown in the bar graphs in Fig. 2 (for exact values see Supplementary Table S1). The DC_50_ values for **5a**–**h** obtained in the buffer containing 20 mM K^+^ indicate that all metallohelices bound preferentially to **22_TERRA** (DC_50_ values ranging from 0.27 to 0.69 µM) versus **26_ds** duplex (DC_50_ values ranging from 0.72 to 1.58 µM). The most potent G-quadruplex binder was **Δ-5b,** followed by **Λ-5b** with the DC_50_ values of 0.27 ± 0.02 and 0.41 ± 0.04 µM, respectively. It should be noted that ligands having DC_50_ values equal to or less than 0.5 µM are regarded as excellent G-quadruplex binders^41^. The selectivity of metallohelices towards the G-quadruplex motif was calculated as a ratio between the DC_50_ values obtained for the **26_ds** and **22_TERRA**. The highest selectivities of 3.6 ± 0.41 and 5.9 ± 0.57 were registered for **Λ-5b** and **Δ-5b**, respectively, while the selectivity values of remaining metallohelices were between 1.3 and 1.7.

Electrostatic interactions play an important role in the binding of highly positively charged metallohelices to the negatively charged backbone of nucleic acids. In order to weaken the electrostatic attraction between the metallohelices and RNA, we performed the FID assays in the presence of increased ionic strength. Inspection of the data in Fig. 2 and Supplementary Table S2 reveals that the DC_50_ values recorded for the binding of **5a**–**h** to the **22_TERRA** in 40 mM K^+^ were slightly increased and the same trend was observed for the **26_ds**. The DC_50_ values of **5a** and **5b** for the **26_ds** were increased markedly more than those of **5c**-**h** and consequently, the binding selectivities of **Λ-5a** and **Δ-5a** towards **22_TERRA** reached values of 3.0 ± 0.2 and 2.9 ± 0.3, respectively, while the binding selectivities of **Λ-5b** and **Δ-5b** were increased even more to 7.9 ± 0.4 and 13.5 ± 0.7, respectively. The selectivities of **5c**–**h** remained almost unchanged in the range of 1.2–1.6. The FID assays for **5a** and **5b** were repeated in the presence of 80 and 160 mM K^+^, which is close to the intracellular concentration of K^+^ (150 mM). Data in Fig. 2 (see also Supplementary Tables S3 and S4) show that the DC_50_ values kept on raising as a function of the concentration of K^+^ and that the DC_50_ values for the **26_ds** raised faster than those for the **22_TERRA**. As a result, the selectivities of **5a** and **5b** were gradually increasing, reaching maximum values of 8 ± 1 and 7 ± 1 for **Λ-5a** and **Δ-5a** and 13 ± 2 and 26 ± 4 for **Λ-5b** and **Δ-5b**, respectively, in the presence of 160 mM K^+^.

### FRET melting assays

**Figure 3:**
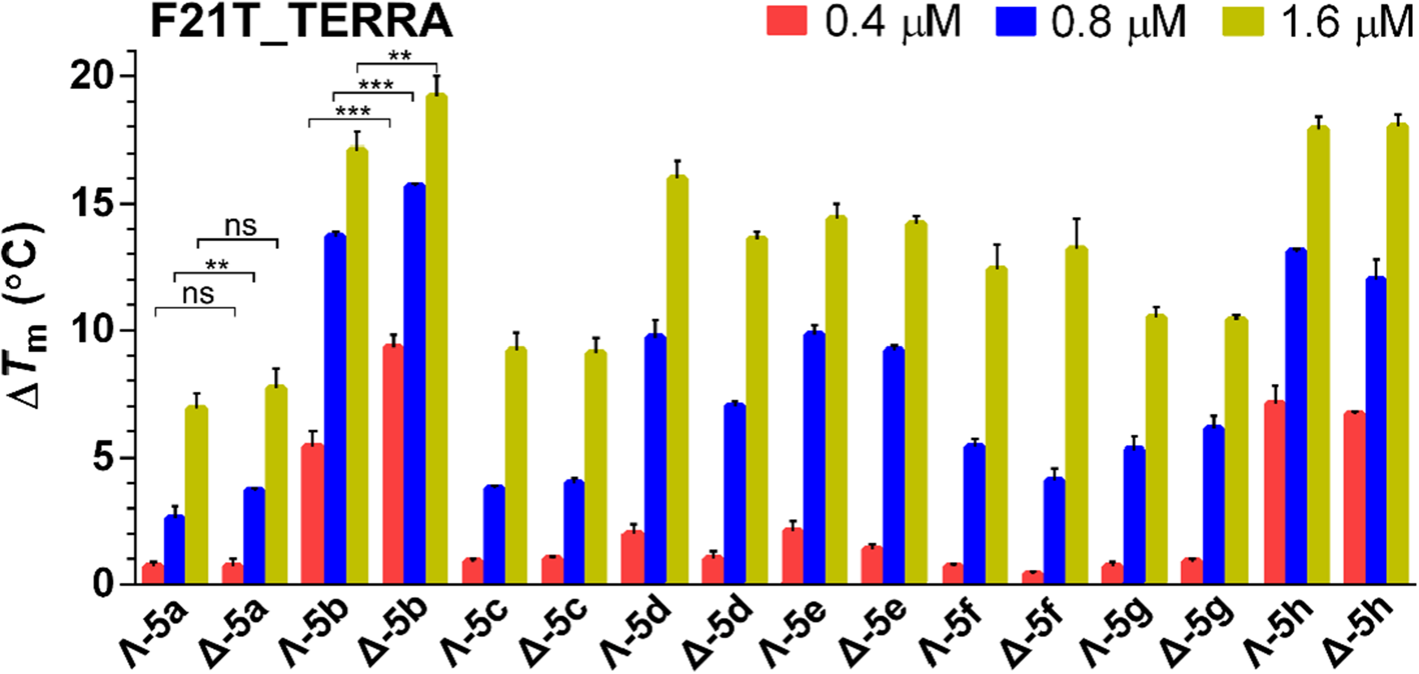
Δ*T*_m_ values for the **F21T_TERRA** (0.4 μM) determined by FRET upon addition of 0.4, 0.8, and 1.6 μM metallohelices. The buffer conditions were 10 mM potassium phosphate (pH 7) and 10 mM KCl. Data shown are expressed as the mean of three independent experiments; error bars indicate the standard error of the mean. Symbols (*, **, ***) and (ns) at the top of the bars indicate statistically significant difference (p ≤ 0.1, p ≤ 0.05, p ≤ 0.01) and statistically insignificant difference (p > 0.1), respectively.

To further investigate the interactions of **5a**–**h** with the RNA G-quadruplex, we employed FRET melting assays. Examples of FRET melting curves obtained for the **F21T_TERRA** G-quadruplex and **F26T_ds** duplex upon the addition of the metallohelices are presented in Supplementary Figures S2 and S3, respectively. The Δ*T*_m_ values for the **F21T_TERRA** summarized in the bar graph in Fig. 3 confirmed the good affinity of **5a**-**h** towards the G-quadruplex motif. It can be seen that the presence of **5a**–**h** at the concentration of 1.6 μM increased the melting temperature of the **F21T_TERRA** by 7–19 °C while the thermal stability of **F26T_ds** (Supplementary Figure S4) was just slightly increased by **5b**-**h** or remained unaffected by **5a**.

**Figure 4:**
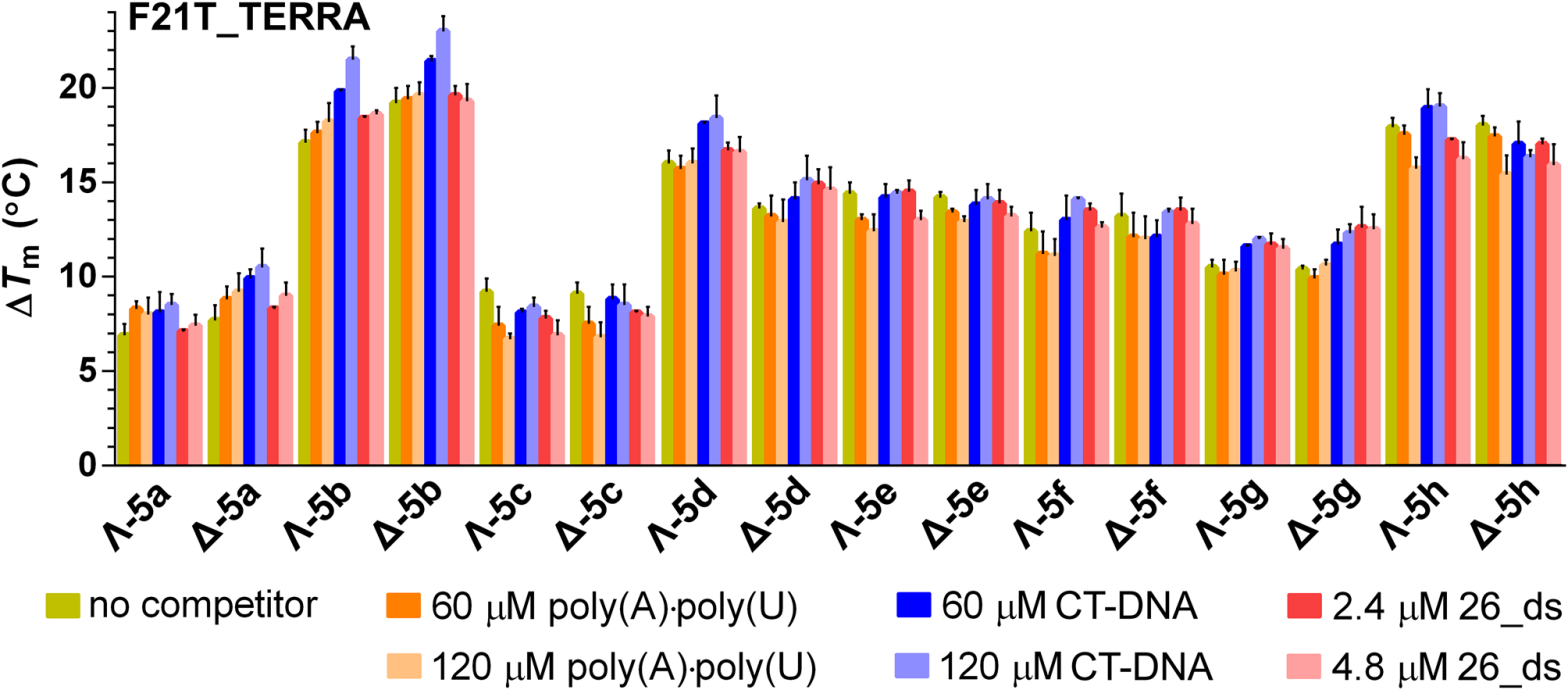
Δ*T*_m_ values for the **F21T_TERRA** (0.4 μM) determined by FRET upon addition of 1.6 μM metallohelices in the presence of various concentrations of poly(A)·poly(U), CT-DNA and **26_ds**. The buffer conditions were 10 mM potassium phosphate (pH 7) and 10 mM KCl. Data shown are expressed as the mean of three independent experiments; error bars indicate the standard error of the mean.

In the next step, we studied the ability of the metallohelices to stabilize the **F21T_TERRA** in the presence of increasing concentrations of competitors; the melting temperature was measured at 1.6 μM concentration of **5a**–**h** in the presence of 60 and 120 μM concentrations (per nucleotide) of synthetic double-stranded polynucleotide poly(A)·poly(U), calf thymus (CT) DNA and **26_ds** (examples of melting curves are shown in Supplementary Figure S5). The Δ*T*_m_ values displayed in Fig. 4 show that the thermal stability of the **F21T_TERRA** in the presence of metallohelices was not markedly affected upon the addition of an excess of competitors. On the other hand, the low stabilizing effect of **5b**–**h** towards the **F26T_ds** was diminished to 0–1 °C upon addition of just 60 μM poly(A)·poly(U) (Supplementary Figure S4). Taken together, these results confirm the binding preference of the metallohelices for G-quadruplex RNA over double-stranded RNA and DNA.

**Figure 5:**
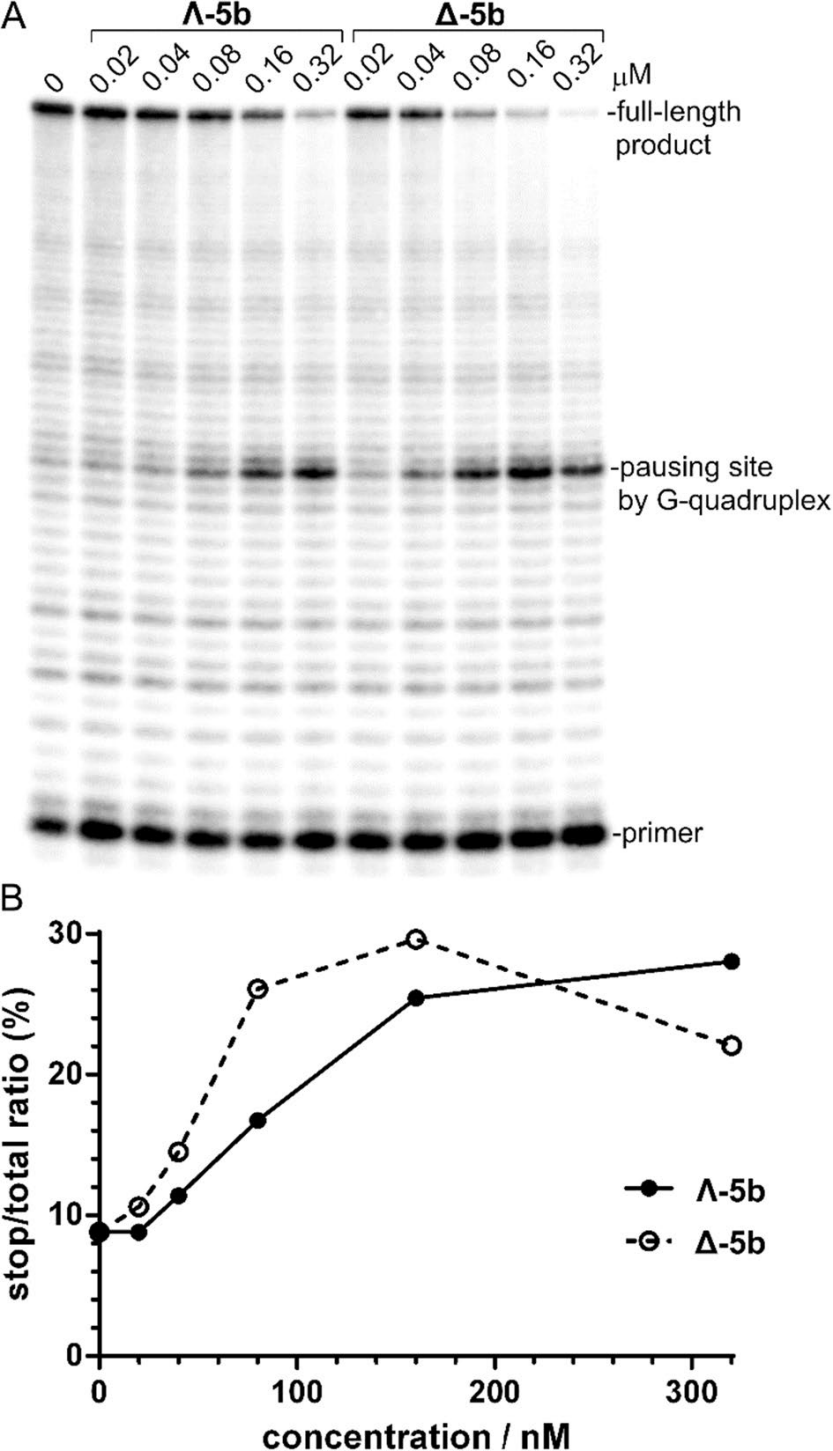
Inhibition of DNA synthesis catalyzed by *ProtoScript* II reverse transcriptase on the G-quadruplex containing **HT4** RNA template (40 nM) at 42 °C in the presence of increasing concentrations (20–320 nM) of **Λ-5b** and **Δ-5b**. (**A**) Autoradiogram of 12% PAA sequencing gel. (**B**) The plot showing the ratio of the radiation corresponding to pausing sites to total radiation of the lane vs concentration of **Λ-5b** and **Δ-5b**.

**Figure 6:**
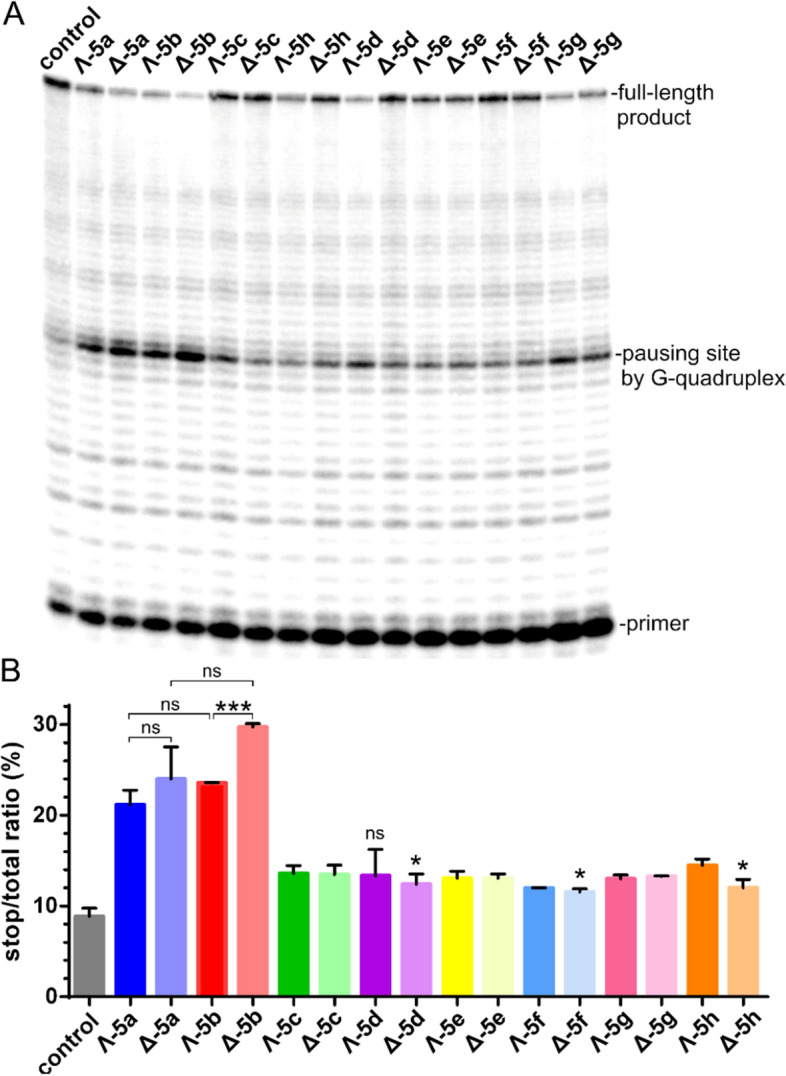
Inhibition of DNA synthesis catalyzed by *ProtoScript* II reverse transcriptase on the G-quadruplex containing **HT4** RNA template (40 nM) at 42 °C in the presence of **5a**–**h** (80 nM). (**A**) Autoradiogram of 12% PAA sequencing gel. (**B**) The bar graph showing the ratio of the radiation corresponding to pausing sites to total radiation of the lane. Data shown are expressed as the mean of two independent experiments; error bars indicate the standard error of the mean. Symbols (*, ***) and (ns) at the top of the bars indicate statistically significant difference (p ≤ 0.1, p ≤ 0.01) and statistically insignificant difference (p > 0.1), respectively. Data obtained for **5a**–**h** were tested against control and only values of p > 0.05 are indicated in the plot.

The reviewer of this manuscript requested to mention in this manuscript that under certain circumstances, the FRET melting assay used to investigate the interactions of the investigated metallohelices with the RNA G-quadruplex may yield, false positive or false negative results. Therefore, we also performed control measurements of the *T*_m_ values using UV melting experiments (see Supplementary Figure S6). However, the UV melting experiments were difficult to evaluate accurately because RNA absorbs at 295 nm only slightly, whereas metallohelices yield in the UV region below 350 nm several narrow and intense peaks (shown for metallohelices **5b** and **5h** in Supplementary Figure S7). Thus, even a relatively small change of the intensity of the peaks and eventual their shifts when the temperature is changed, have a high impact on the shape of the melting curves recorded at 295 nm, which makes their analysis more difficult. Additionally, the less accurate evaluation of the UV melting curves shown in Supplementary Figure S6 nevertheless confirms the conclusions drawn on the basis of the results of FRET melting experiments which were also confirmed by the results of the experiments in which the fluorescent intercalator displacement (FID) assay (Fig. 2) and Reverse transcriptase stop assay (Figs. 5 and 6) were used. It is also important to emphasize that FRET melting assay has been widely used to evaluate the stability of G-quadruplexes in the presence of various ligands, see Refs.^42,43^ as examples.

### Reverse transcriptase stop assay

The following assay was employed to determine whether **5a**–**h** interfere with the DNA synthesis catalyzed by reverse transcriptase (RT) on RNA template containing four repeats of the human telomeric sequence. It is a modification of a DNA polymerase arrest assay developed by Han et al.^44^ for DNA G-quadruplex-interactive ligands. We used *ProtoScript* II RT which is a recombinant M-MuLV RT with reduced RNase H activity and increased thermostability. The results of primer extension reactions catalyzed by *ProtoScript* II RT on the **HT4** RNA template (for its sequence see “Experimental section”) in the presence of increasing concentrations of **Λ-5b** and **Δ-5b** at 42 °C are shown in Fig. 5.

As can be seen in the autoradiogram of the gel, the presence of a G-rich site in the RNA template led to slight pausing of the RT. The addition of **Λ-5b** and **Δ-5b** caused an enhancement of pausing at the same site as that observed in the absence of metallohelices suggesting that both enantiomers increase pausing of the RT by stabilizing the G-quadruplex structure. The results were quantified and the plot in Fig. 5B reveals that both enantiomers are able to inhibit DNA polymerization at sub-micromolar concentrations and that **Δ-5b** is a slightly more efficient inhibitor than **Λ-5b,** which is in agreement with the previous results.

In order to compare the potency of **5a**–**h** to inhibit the activity of RT, we performed the RT stop assay for all metallohelices at a fixed concentration of 80 nM (Fig. 6). The results presented in the bar graph in Fig. 6B demonstrate that all metallohelices interfere to some extent with RT activity by stabilizing RNA G-quadruplex, but as expected **Δ-5b** was the most potent inhibitor followed by **Λ-5b** and both enantiomers of **5a**.

### RNase A digestion

In an effort to characterize the interaction of **Λ-5b** and **Δ-5b** with the **22_TERRA**, we performed mapping experiment using RNase A. This endoribonuclease preferentially cuts single- over double-stranded RNA at the 3′-end of unpaired C and U residues making it a useful tool for investigating drug binding to the single-stranded regions of the RNA such as loops and bulges. The RNase A degradation pattern of 3′-^32^P end-labeled **22_TERRA** is shown in Supplementary Figure S8, and as it can be observed (see also plots in Supplementary Figure S9), the cleavage at residues U17 and U11 was unchanged or only slightly enhanced at the doses of **Λ-5b** and **Δ-5b** corresponding to the metallohelix:G-quadruplex ratio 1:1–4:1. The presence of low concentrations of the metallohelices corresponding to the metallohelix:G-quadruplex ratio 1:1–2:1 had little effect on the degradation at residue U5, however when the metallohelix:G-quadruplex ratio exceeded 2:1 the reduction of RNase A cleavage was more pronounced (Supplementary Figure S9).

The low impact of **5b** at 1:1 and 2:1 metallohelix:G-quadruplex ratios on the RNase A cleavage at residues U5, U11, and U17 located on the 3′-end of the G-quadruplex suggests that both enantiomers may bind by external stacking to the G-quartet on the opposite 5′-end of the G-quadruplex structure (see scheme in Fig. 1B).

### 2-aminopurine (2Ap) fluorescence studies

To further evaluate the interaction of **5a**–**h** with the human telomeric RNA G-quadruplex, we employed **22_TERRA** labeled by 2-aminopurine (2Ap) at positions 7, 13, and 19 (see scheme in Fig. 1B). It was verified by CD spectroscopy (Supplementary Figure S10) that 2Ap modified **22_TERRA** had the same conformation as the wild type. 2Ap, the fluorescent analog of adenine, has been often used to probe the binding of small molecules to DNA and RNA^45,46^. The fluorescence intensity of 2Ap is efficiently quenched within the structure of double-stranded DNA and RNA, but it is enhanced when the base stacking or base pairing is perturbed. Other factors that affect 2Ap fluorescence are collisions with other bases and biomolecular interactions^47,48^. The 2Ap-labeled **22_TERRA** was first titrated with **5a** and **5b** in a series of experiments to select an appropriate metallohelix:RNA quadruplex ratio (Supplementary Figure S11). The plots show that both metallohelices efficiently reduced 2Ap fluorescence and that **5b** was slightly more efficient than **5a**. The intensity of 2Ap fluorescence was then measured at a fixed concentration of 2Ap-labeled **22_TERRA** (2.5 μM) and two different concentrations (2.5 and 5 μM) of metallohelices corresponding to 1:1 and 2:1 metallohelix:G-quadruplex ratios. Results obtained for the 1:1 and 2:1 metallohelix:G-quadruplex ratios are displayed as bar graphs in Supplementary Figures S12 and S13, respectively. As it can be seen in both Figures, **5a**–**h** quenched the fluorescence of 2Ap regardless of its position in the RNA G-quadruplex. **Λ-5b** and **Δ-5b** were the most potent quenchers of 2Ap fluorescence, followed by the enantiomers of **5a** and **5d**. The differences between Λ- and Δ-enantiomers were small, however, **Δ-5b** was slightly more efficient quencher than **Λ-5b**. One can notice that **5h** was markedly less active than other metallohelices in reducing the fluorescence of 2Ap, but in this case, the results are influenced by the intrinsic fluorescence of **5h** (see Supplementary Figure S14), which interferes with the fluorescence of 2Ap at 365 nm.

The nearly equal reduction of the fluorescence of 2Aps located in the loops at positions 7, 13, and 19 at both 1:1 and 2:1 metallohelix:G-quadruplex ratios suggests that **5a**–**h** bind via external stacking to the terminal G-quartet on the 5′-side of the G-quadruplex as it has been proposed by RNase A footprinting. The binding of metallohelices might induce such conformational changes that enhance stacking interactions of 2Aps with adjacent bases and increase the efficacy of the electron transfer quenching. It is also possible that the quenching action of the metallohelices originates from direct interactions of **5a**–**h** with 2Aps.

One aspect of the binding mode of some ligands stabilizing DNA or RNA G quadruplexes is unfolding, or conformational changes of G-quadruplexes due to the binding of ligands and an efficient and widely used method to investigate these aspects of the binding mode is CD spectroscopy. The analysis of CD spectra of the mixture of **22_TERRA** with metallohelices below 300 nm could not be interpreted in terms of the structural changes in the RNA G-quadruplex because the metallohelices also yield a very strong CD signal in the region of wavelengths in which RNA strongly absorbs (Supplementary Figure S15). Thus, interpretation of the CD spectra of the mixtures of the metallohelices and RNA in terms of conformational changes induced by metallohelices in RNA G quadruplexes is impossible.

## Conclusions

In this study, we demonstrate that metallohelices **5a**–**h** bind with a high affinity to TERRA G-quadruplex and that their binding preferences for the G-quadruplex structure over double-stranded (ds) RNA differ considerably across the range of structures. Enantioselective recognition was observed for some of the metallohelices, particularly **5a** and **5b**. **Δ-5b** was shown to be the most potent G-quadruplex binder followed by **Λ-5b** and by the enantiomers of **5a**. Interestingly, both enantiomers of **5b** exhibited also the highest affinity towards various DNA G-quadruplexes including one formed from the human telomeric sequence. Metallohelices **5c**–**h** also preferred binding to TERRA G-quadruplex over dsRNA but their binding selectivity was much lower, as compared to **5b** and **5a**.

The higher binding affinity of **5a** and its *meta* analog **5b** to TERRA G-quadruplex might be associated with different dimensions and overall shape of these two metallohelices compared to **5c**-**h**. **5b** has the shortest intermetallic distance of 12.4 Å, followed by **5a** and **5h** with 14 Å and 14.4 Å, respectively. The intermetallic distances in **5c**–**g** are markedly longer > 17 Å. The shorter Fe–Fe distance provides **5a** and **5b** with higher charge density and gives these compounds a more compact shape which seems to be more favorable for the binding to G-quadruplexes and less favorable for the binding to double-helical RNA. The longer metallohelices **5c**–**h** exhibit higher affinity towards double-helical RNA than **5a** and **5b** which leads to their lower binding selectivities against G-quadruplexes.

The binding selectivity of **5a** and particularly **5b** to the TERRA G-quadruplex increases with increasing ionic strength. This is because the presence of cations weakened the binding of **5a** and **5b** to dsRNA more than to the G-quadruplex structure. It suggests that the electrostatic forces are mainly involved in the interaction of **5a** and **5b** with dsRNA and play a less important role in binding to the G-quadruplex. On the contrary, the binding affinities of longer metallohelices **5c**–**h** to the G-quadruplex and dsRNA were affected to a similar degree by the presence of a higher concentration of K^+^ which resulted in a negligible effect of the ionic strength on the binding selectivities. It seems that the binding of **5c**–**h** to dsRNA is not predominantly driven by the electrostatic forces or that the strengthening of another kind of interaction between the metallohelix and the double-helix compensates for the suppression of the electrostatic attraction in high salt concentration.

The presence of just 80 nM concentration of **5a**–**h** was shown to inhibit DNA synthesis on the RNA template containing four repeats of the human telomeric sequence by stabilizing the G-quadruplex structure. The highest inhibitory effect was observed for **Δ-5b,** followed by **Λ-5b** and both enantiomers of **5a.**

The binding mode of the **5a**–**h** was not fully resolved, however, the results from the experiments with 2Ap-labeled G-quadruplex and the cleavage by RNase A suggest that the metallohelices stack externally to the terminal G-quartet on the 5′-side of the TERRA G-quadruplex. Stacking interaction with the terminal G-quartet has been previously proposed for the binding of **5a** with human telomeric DNA G-quadruplex^34^. In summary, our results show that, **5a**–**h** metallohelices are potent stabilizers of TERRA G-quadruplex and act as inhibitors of reverse transcription on the template containing four repeats of human telomeric sequence. If we take into account that metallohelices are able to accumulate inside cells and in the nucleus, we can speculate that stabilization of RNA sequences capable of G-quadruplex formation by metallohelices might contribute to the mechanism of their biological activity.

## Experimental section

### Chemicals and reagents

The metallohelices **5a**–**5h** were synthesized and characterized, as described previously^36^. The synthetic oligoribonucleotides and oligodeoxyribonucleotides used in this work were purchased from Eurofins Genomics (Ebersberg, Germany). T4 polynucleotide kinase and ProtoScript II reverse transcriptase were purchased from New England Biolabs (Beverly, MA). Calf thymus DNA (CT-DNA), poly(A)·poly(U), thiazole orange (TO) and RNase A were from Sigma-Aldrich (Prague, Czech Republic). [γ-^32^P]-ATP was from Hartmann analytic GmbH (Braunschweig, Germany). Acrylamide and bis(acrylamide) were from Merck KgaA (Darmstadt, Germany).

### FID measurements

Oligoribonucleotides **22_TERRA**, 5′-AGGGUUAGGGUUAGGGUUAGGG-3′ and **26_ds** hairpin 5′-CAAUCGGAUCGAAUUCGAUCCGAUUG-3′ (6.25 μM) were annealed in 10 mM potassium phosphate buffer (pH 7) and in the presence of various concentrations of KCl by heating to 85 °C for 3 min followed by slow cooling to room temperature. Thiazole orange solutions (10 mM) in DMSO were prepared fresh each week. Oligoribonucleotides (0.25 µM) were mixed with 0.5 µM thiazole orange in a 1 cm quartz cuvette in a total volume of 2.5 mL and titrated with metallohelices. Small aliquots (typically 2.5 µL) of concentrated solutions (typically 1 × 10^–4^ M) of metallohelices were added to the mixture. Samples were vigorously mixed by pipetting and left to equilibrate for 3 min at room temperature before data taking. Measurements were performed with the Varian Cary Eclipse spectrofluorophotometer using the following parameters: the excitation and emission wavelengths were set to 501 nm and 538 nm, respectively, widths of the excitation and emission slit were 10 nm, and the averaging time was set to 3 s.

### FRET melting assays

The solutions of the fluorescent-labeled (donor fluorophore FAM, 6-carboxyfluorescein; acceptor fluorophore TAMRA, 6-carboxytetramethylrhodamine) oligoribonucleotides **F21T_TERRA**, 5′-FAM-GGGUUAGGGUUAGGGUUAGGG-TAMRA-3′, and **F26T_ds** hairpin, 5′-FAM-CAAUCGGAUCGAAUUCGAUCCGAUUG-TAMRA-3′ (4 µM) were annealed in 10 mM potassium phosphate buffer (pH 7) and 10 mM KCl by heating to 85 °C for 3 min followed by slow cooling to room temperature. Oligoribonucleotides (0.4 μM) were either mixed with 0.4, 0.8, and 1.6 μM metallohelices in the absence of the competitors or were added at the concentration of 1.6 μM in the presence of various concentrations of poly(A)·poly(U), CT-DNA or **26_ds** hairpin. Samples were prepared in a total volume of 40 µL in 0.2 mL microtubes. Measurements were carried out using the real-time PCR instrument RotorGene 6000 (Corbett Research). The excitation and detection wavelengths were set to 470 ± 10 nm and 510 ± 5 nm, respectively, and the temperature was raised at a rate of 0.7 °C/min. The data readings were taken every 60 s. The melting temperatures (*T*_m_) were determined from the inflection point of the melting curves by applying a first derivative calculation using the RotorGene 6000 application software.

### Reverse transcriptase (RT) stop assay

DNA primer **P18**, 5′-TAATACGACTCACTATAG-3′ (40 nM) was 5′-end labeled with [γ-^32^]ATP using T4 polynucleotide kinase and mixed with RNA template **HT4**, 5′-UCCAACUAUGUAUACUUAGGGUUAGGGUUAGGGUUAGGGACAUAUCGAUGAAAUUGCUAUAGUGAGUCGUAUUA-3′ (40 nM) in 10 mM Tris–HCl (pH 8) buffer containing 1.5 mM MgCl_2_, 35 mM NaCl and 5 mM KCl. The mixture was denatured by heating to 85 °C and allowed to cool down to room temperature. Metallohelices at various concentrations were added to the mixture (10 μL final volume) and incubated at room temperature for 10 min. The primer extension reactions were initiated by adding 0.25 mM dNTPs, 10 mM dithiothreitol (DTT) and 10 units of *ProtoScript II* RT (recombinant M-MuLV reverse transcriptase with reduced RNase H activity and increased thermostability). After incubation at 42 °C for 60 min, the reactions were stopped by adding an equal volume of 2 × concentrated formamide loading buffer and the products were separated on a 12% PAA sequencing gel.

### RNase A digestion

**22_TERRA** (the same oligoribonucleotide as for FID measurements) was 5′-end labeled using T4 polynucleotide kinase and [γ-^32^P]ATP and annealed by heating at 85 °C for 3 min, followed by slow cooling to room temperature in 10 mM sodium phosphate buffer (pH 7), 25 mM NaCl and 5 mM KCl. 4 μL solutions containing 2 × 10^–6^ M **22_TERRA** and various concentrations of the metallohelices were incubated for 10 min at room temperature. Cleavage was initiated by the addition of 1 μL (~ 6 × 10^–6^ units) of RNase A. Samples were incubated for 12 min at room temperature before quenching with 5 μL of 2 × concentrated formamide loading buffer followed by heating at 90 °C for 3 min. RNA cleavage products were resolved by PAA gel electrophoresis under denaturing conditions (24%/8 M urea).

### 2-aminopurine (2Ap) fluorescence studies

The solutions of 2Ap-labeled oligoribonucleotides **22_TERRA_2Ap7**, 5′-AGGGUU*2Ap*GGGUUAGGGUUAGGG-3′, **22_TERRA_2Ap13**, 5′-AGGGUUAGGGUU*2Ap*GGGUUAGGG-3′, and **22_TERRA_2Ap19**, 5′-AGGGUUAGGGUUAGGGUU*2Ap*GGG-3′ (40 μM) were annealed in 10 mM potassium phosphate (pH 7.0) and 10 mM KCl by heating to 85 °C for 3 min followed by slow cooling to room temperature. Oligoribonucleotides (2.5 μM) were mixed with 2.5 μM or 5 μM metallohelix in 10 mM potassium phosphate (pH 7.0) and 10 mM KCl in a total volume of 80 μL and placed in a quartz micro cuvette (volume of 50 μL). Samples were left undisturbed at room temperature for 5 min before taking measurement. Oligoribonucleotides (2.5 μM) were titrated with metallohelices in a 0.5 × 0.5 cm quartz cuvette in a total volume of 0.6 mL in 10 mM potassium phosphate (pH 7.0) and 10 mM KCl. Samples were thoroughly mixed by pipetting and kept undisturbed for 3 min at room temperature before data taking. Measurements were carried out using the Varian Cary Eclipse spectrofluorophotometer and the following parameters: the excitation and emission wavelengths were set to 310 nm and 365 nm, respectively, widths of the excitation and emission slit were 10 nm, and the averaging time was set to 3 s.

### Other physical methods

UV absorbance measurements were conducted on a Varian Cary 4000 UV/vis spectrophotometer. Gels containing radioactively labeled samples with [γ-^32^P]ATP were exposed to a phosphor imaging plate and scanned with a GE Healthcare FLA 7000 laser scanner.

## Supplementary information

Supplementary information.
