# Supplementary material for: Stabilization of human telomeric RNA G-quadruplex by the water-compatible optically pure and biologically-active metallohelices
Source: Sci Rep. 2020 Sep 3;10:14543. doi: 10.1038/s41598-020-71429-5 (PMC7471899; doi:10.1038/s41598-020-71429-5)
Supplement: Supplementary file 1 — Supplementary information. [file 41598_2020_71429_MOESM1_ESM.pdf]

Supporting Information

for

Stabilization of human telomeric RNA G-quadruplex by the water-compatible optically pure and biologically-active metallohelices

Jaroslav Malina[1], Peter Scott[2] and Viktor Brabec[1]✉

[1]Czech Academy of Sciences, Institute of Biophysics, Kralovopolska 135, CZ-61265 Brno, Czech Republic
[2]Department of Chemistry, University of Warwick, Gibbet Hill Road, Coventry, CV4 7AL, UK

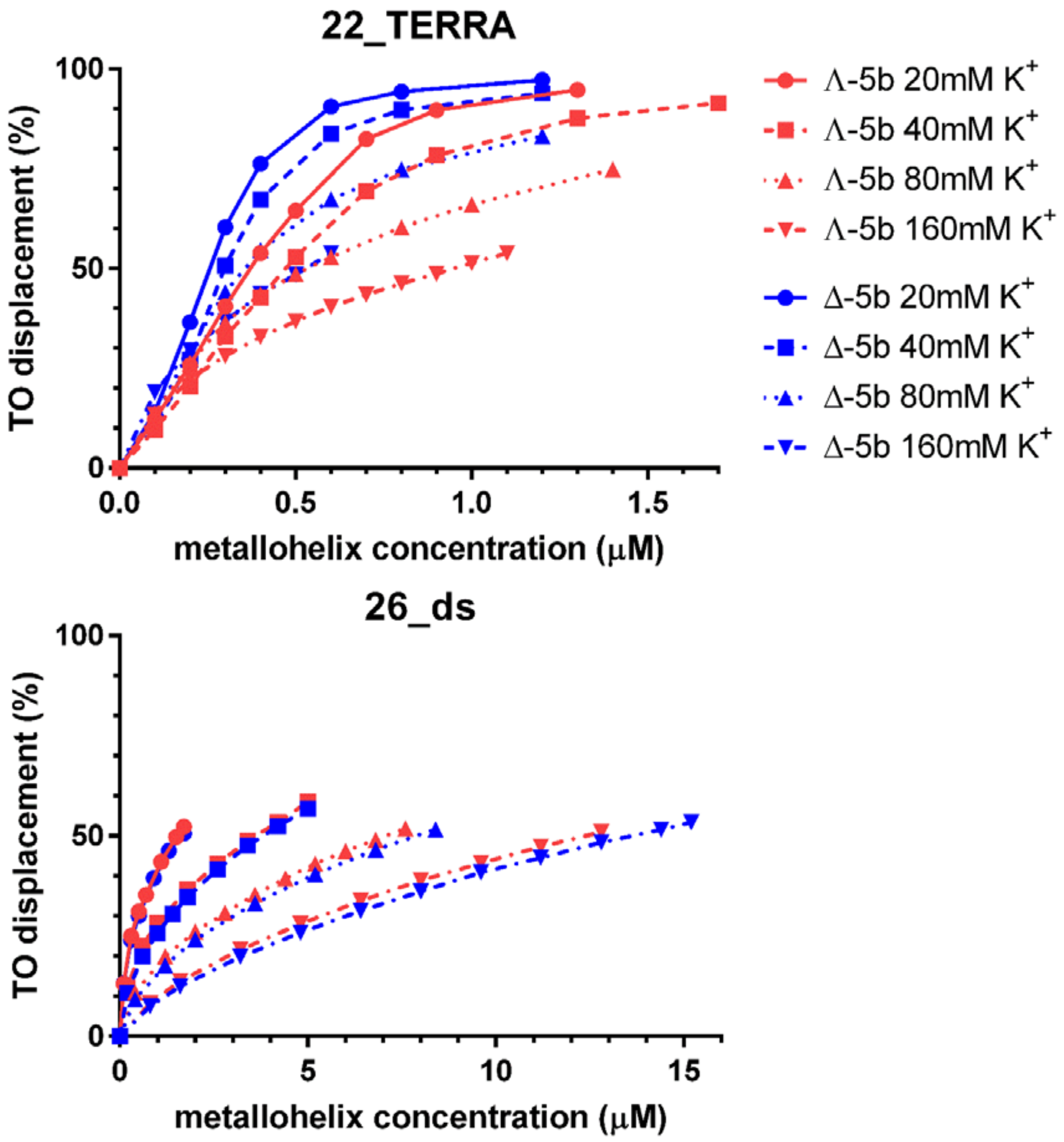

Figure S1. Thiazole orange (TO) displacement from **22_TERRA** (upper panel) and **26_ds** by Λ- and Δ-enantiomers of **5b.** The titrations were conducted in 10 mM potassium phosphate (pH 7) and various concentrations of KCl (10, 30, 70, and 150 mM).

Table S1. DC_{50} values (μM) for **22_TERRA** and **26_ds** in 10 mM KCl and 10 mM potassium phosphate buffer (pH 7) determined by FID upon addition of metallohelices

**Compound**	**TO displacement (DC_{50})**		**Selectivity (DC_{50})**
	**22_TERRA**	**26_ds**	**26_ds/ 22_TERRA**
**Λ-5a**	0.47 ± 0.05	0.72 ± 0.02	1.5 ± 0.17
**Δ-5a**	0.57 ± 0.06	0.95 ± 0.08	1.7 ± 0.22
**Λ-5b**	0.41 ± 0.04	1.47 ± 0.09	3.6 ± 0.41
**Δ-5b**	0.27 ± 0.02	1.58 ± 0.10	5.9 ± 0.57
**Λ-5c**	0.59 ± 0.04	0.83 ± 0.04	1.4 ± 0.12
**Δ-5c**	0.69 ± 0.06	0.90 ± 0.07	1.3 ± 0.15
**Λ-5d**	0.59 ± 0.01	0.76 ± 0.03	1.3 ± 0.06
**Δ-5d**	0.61 ± 0.02	0.82 ± 0.03	1.3 ± 0.07
**Λ-5e**	0.68 ± 0.03	0.91 ± 0.04	1.3 ± 0.08
**Δ-5e**	0.66 ± 0.02	0.93 ± 0.03	1.4 ± 0.06
**Λ-5f**	0.47 ± 0.05	0.72 ± 0.07	1.5 ± 0.22
**Δ-5f**	0.60 ± 0.06	0.80 ± 0.05	1.3 ± 0.16
**Λ-5g**	0.51 ± 0.03	0.78 ± 0.04	1.5 ± 0.12
**Δ-5g**	0.57 ± 0.05	0.77 ± 0.05	1.4 ± 0.15
**Λ-5h**	0.63 ± 0.04	0.87 ± 0.06	1.4 ± 0.13
**Δ-5h**	0.64 ± 0.05	0.89 ± 0.02	1.4 ± 0.11

Table S2. DC_{50} values (μM) for **22_TERRA** and **26_ds** in 30 mM KCl and 10 mM potassium phosphate buffer (pH 7) determined by FID upon addition of metallohelices

**Compound**	**TO displacement (DC_{50})**		**Selectivity (DC_{50})**
	**22_TERRA**	**26_ds**	**26_ds/ 22_TERRA**
**Λ-5a**	0.52 ± 0.02	1.55 ± 0.06	3.0 ± 0.16
**Δ-5a**	0.62 ± 0.05	1.82 ± 0.09	2.9 ± 0.28
**Λ-5b**	0.46 ± 0.02	3.65 ± 0.06	7.9 ± 0.37
**Δ-5b**	0.30 ± 0.01	4.05 ± 0.15	13.5 ± 0.67
**Λ-5c**	0.61 ± 0.01	0.83 ± 0.05	1.4 ± 0.09
**Δ-5c**	0.68 ± 0.02	0.88 ± 0.04	1.3 ± 0.07
**Λ-5d**	0.64 ± 0.03	0.81 ± 0.05	1.3 ± 0.10
**Δ-5d**	0.62 ± 0.02	0.78 ± 0.03	1.3 ± 0.06
**Λ-5e**	0.71 ± 0.02	0.87 ± 0.05	1.2 ± 0.08
**Δ-5e**	0.67 ± 0.03	0.86 ± 0.06	1.3 ± 0.11
**Λ-5f**	0.54 ± 0.02	0.76 ± 0.04	1.4 ± 0.09
**Δ-5f**	0.66 ± 0.02	0.78 ± 0.03	1.2 ± 0.06
**Λ-5g**	0.56 ± 0.03	0.77 ± 0.05	1.4 ± 0.12
**Δ-5g**	0.61 ± 0.04	0.80 ± 0.04	1.3 ± 0.11
**Λ-5h**	0.67 ± 0.03	0.96 ± 0.05	1.4 ± 0.10
**Δ-5h**	0.64 ± 0.05	1.00 ± 0.06	1.6 ± 0.15

Table S3. DC_{50} values (μM) for **22_TERRA** and **26_ds** in 70 mM KCl and 10 mM potassium phosphate buffer (pH 7) determined by FID upon addition of metallohelices.

**Compound**	**TO displacement (DC_{50})**		**Selectivity (DC_{50})**
	**22_TERRA**	**26_ds**	**26_ds/ 22_TERRA**
**Λ-5a**	0.61 ± 0.08	3.48 ± 0.15	5.7 ± 0.79
**Δ-5a**	0.71 ± 0.10	3.98 ± 0.12	5.6 ± 0.81
**Λ-5b**	0.55 ± 0.02	7.07 ± 0.21	12.9 ± 0.60
**Δ-5b**	0.37 ± 0.04	7.88 ± 0.25	21 ± 2.4

Table S4. DC_{50} values (µM) for **22_TERRA** and **26_ds** in 150 mM KCl and 10 mM potassium phosphate buffer (pH 7) determined by FID upon addition of metallohelices.

Compound	TO displacement (DC_{50})		Selectivity (DC_{50})
	22_TERRA	26_ds	26_ds/ 22_TERRA
**Λ-5a**	0.88 ± 0.11	7.15 ± 0.45	8 ± 1.1
**Δ-5a**	1.12 ± 0.13	8.36 ± 0.60	7 ± 1
**Λ-5b**	0.95 ± 0.15	12.4 ± 0.5	13 ± 2.1
**Δ-5b**	0.53 ± 0.08	13.7 ± 0.6	26 ± 4.1

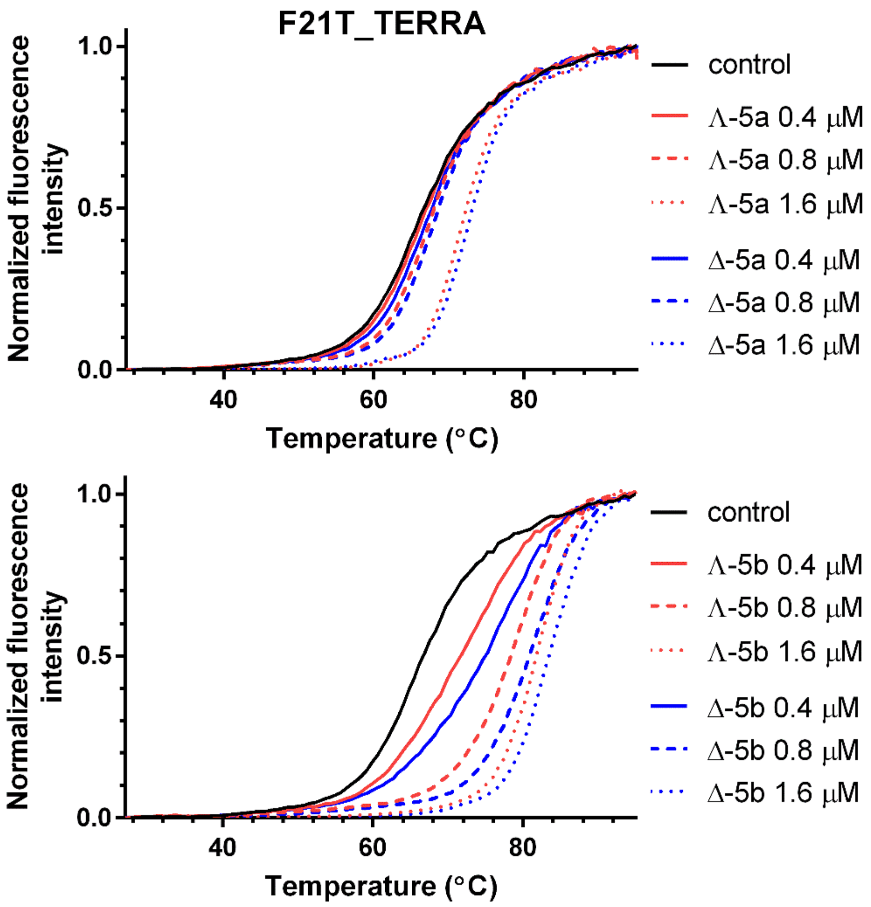

Figure S2. FRET melting curves for **F21T_TERRA** (0.4 µM) in the presence of various concentrations of **Λ-5a**, **Δ-5a**, **Λ-5b** and **Δ-5b**. The buffer conditions were 10 mM potassium phosphate (pH 7) and 10 mM KCl.

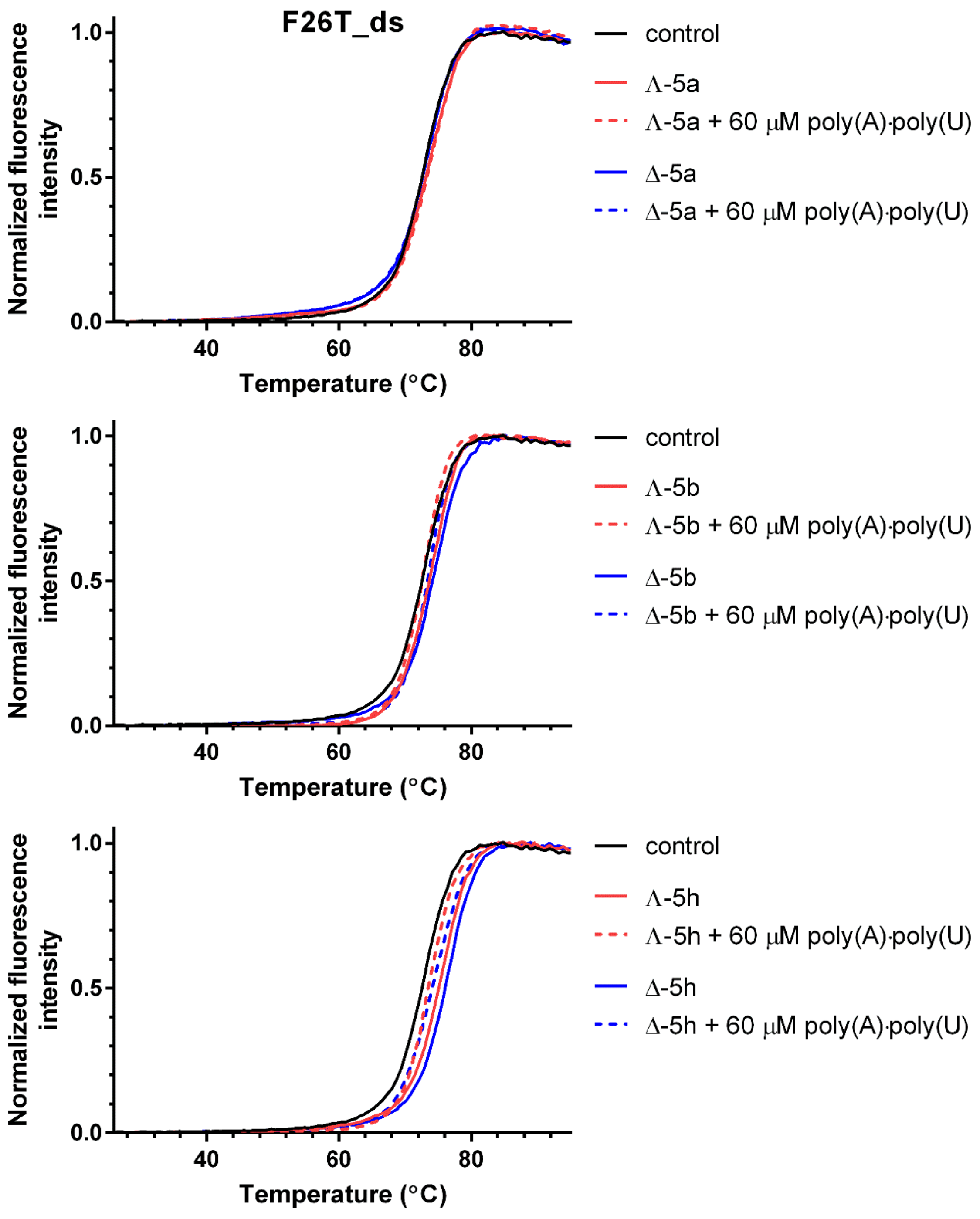

Figure S3. FRET melting curves for **F26T_ds** (0.4 μM) upon addition of Λ- and Δ-enantiomers of **5a**, **5b** and **5h** (1.6 μM) in the absence and in the presence of 60 μM poly(A)·poly(U). The buffer conditions were 10 mM potassium phosphate (pH 7) and 10 mM KCl.

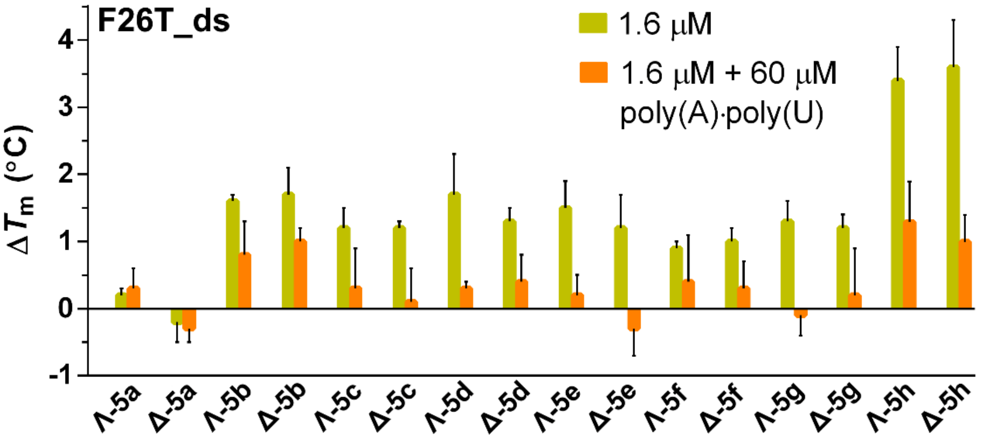

Figure S4. ΔT_m values for **F26T_ds** determined by FRET upon addition of metallohelices. **F26T_ds** (0.4 μM) was mixed with 1.6 μM metallohelices in the absence and in the presence of 60 μM poly(A)·poly(U). The buffer conditions were 10 mM potassium phosphate (pH 7) and 10 mM KCl. Data shown are expressed as the mean of three independent experiments; error bars indicate the standard error of the mean.

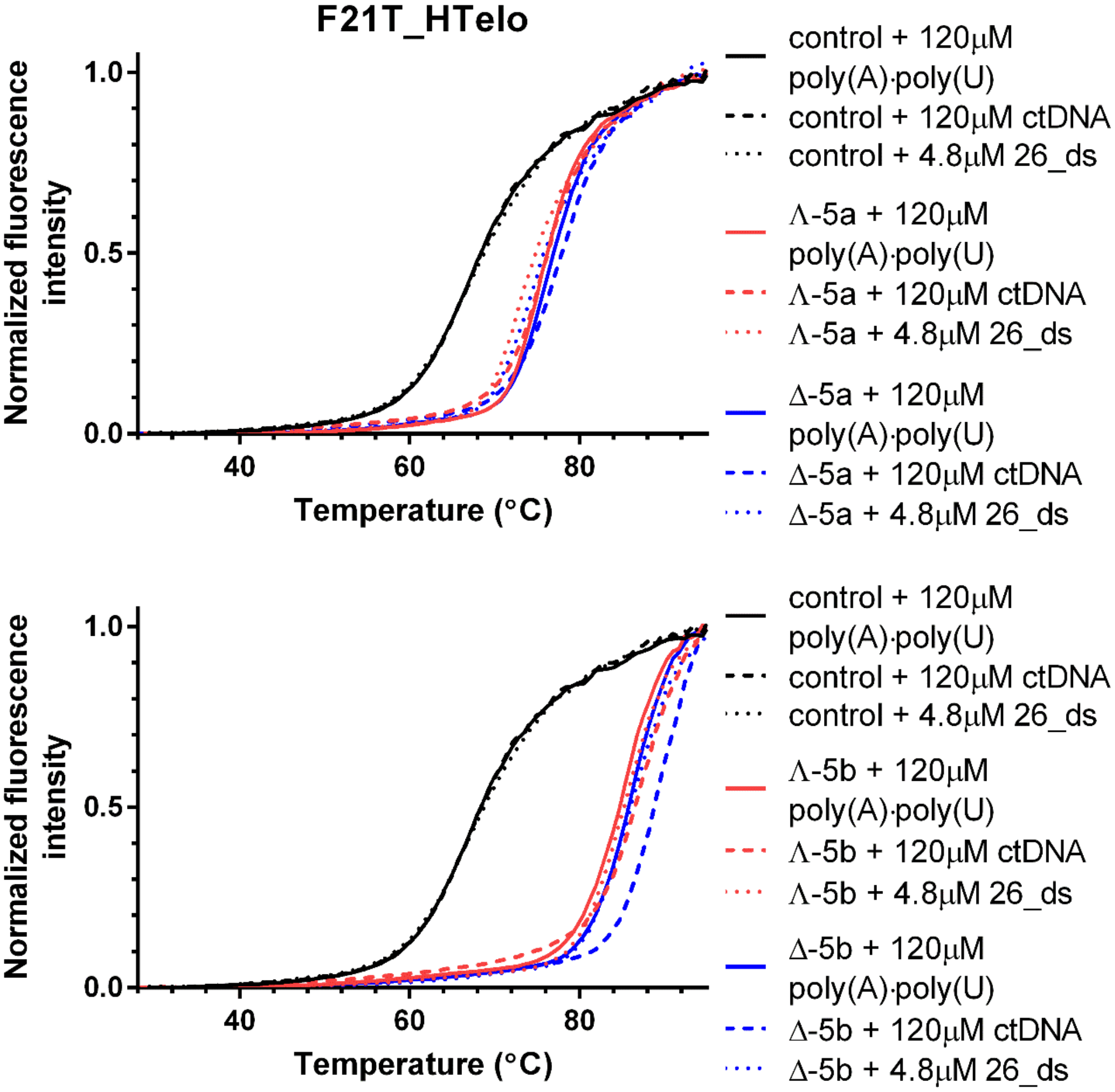

Figure S5. FRET melting curves for **F21T_TERRA** (0.4 μM) upon addition of **Λ-5a**, **Δ-5a**, **Λ-5b** and **Δ-5b** (1.6 μM) and in the presence of 120 μM poly(A)·poly(U), 120 μM CT-DNA or 4.8 μM **26_ds**. The buffer conditions were 10 mM potassium phosphate (pH 7) and 10 mM KCl.

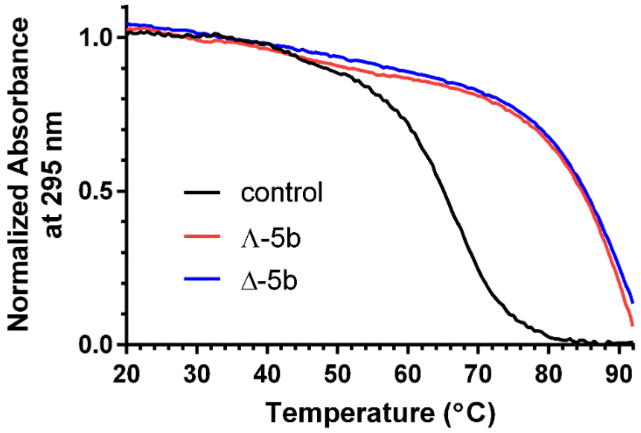

Figure S6. Normalized UV melting curves of **22_TERRA** (2.5 µM) in the absence and in the presence of **5b** (5 µM). UV melting curves were recorded at 295 nm and the buffer conditions were 10 mM potassium phosphate (pH 7) and 10 mM KCl.

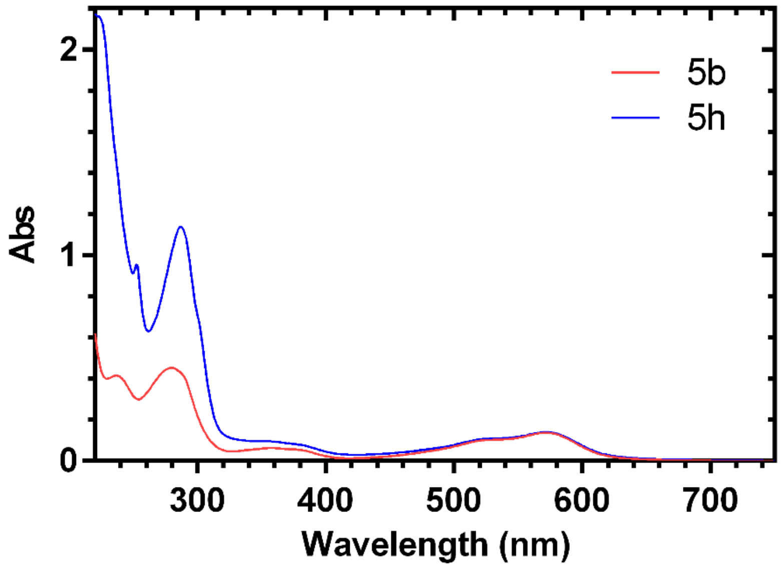

Figure S7. Absorption spectra of of **5b** and **5h** at the concentration of 10 µM in water.

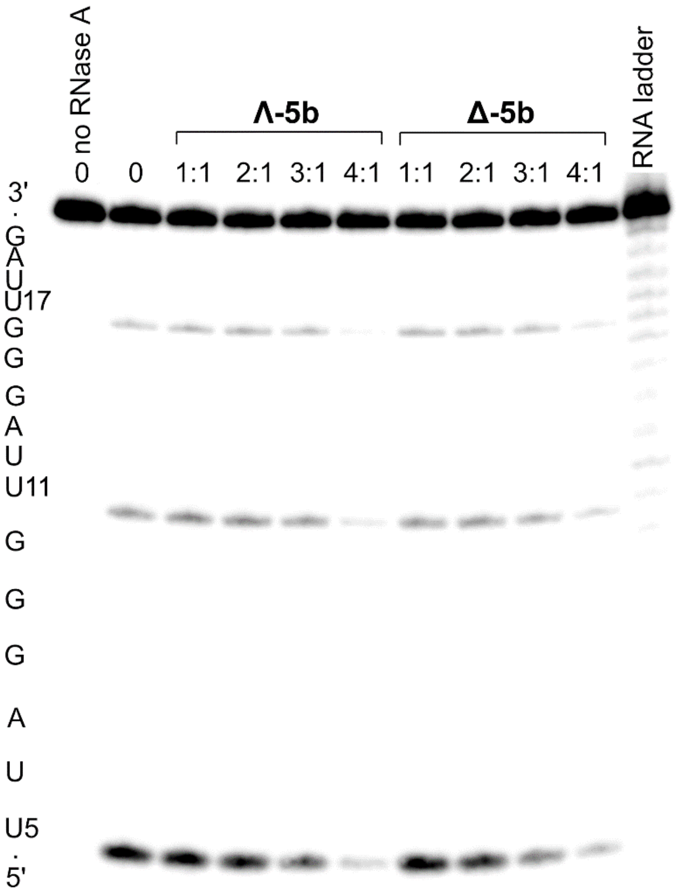

Figure S8. Autoradiogram of 24% PAA sequencing gel with products of RNase A cleavage of 5'-^{32}P end-labelled **22_TERRA** (2 μM) in the presence of increasing concentrations of **Λ-5b** and **Δ-5b**. **22_TERRA** was mixed with **5b** at 1:1, 2:1, 3:1, and 4:1 (metallohelix:oligo) ratios, respectively. The buffer conditions were 10 mM sodium phosphate buffer (pH 7), 25 mM NaCl and 5 mM KCl.

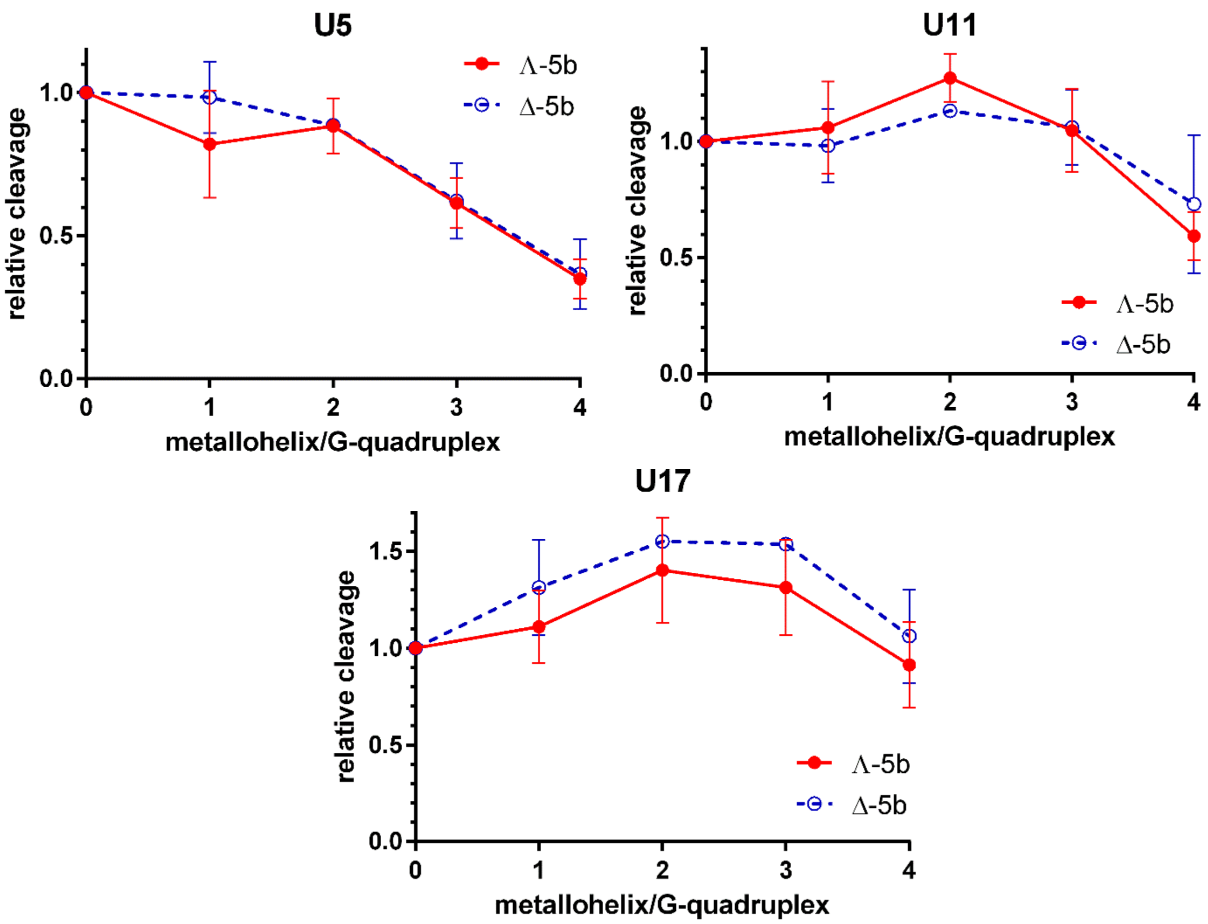

Figure S9. RNase A cleavage of 5‘-^{32}P end-labelled **22_TERRA** (2 µM) in the presence of increasing concentrations of **Λ-5b** and **Δ-5b**. The plots show the ratio of radiation corresponding to cleavage sites at positions U5, U11 and U17 to total radiation of the lane *vs* concentration of the metallohelices. Data shown are expressed as the mean of two independent experiments; error bars indicate the standard error of the mean.

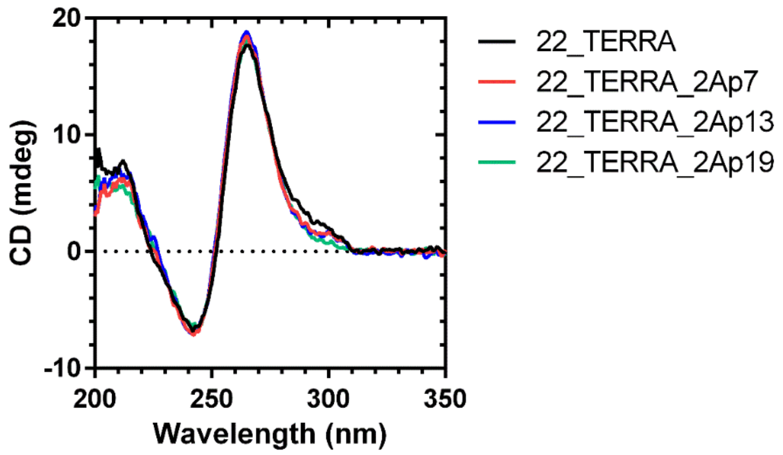

Figure S10. CD spectra of 22_TERRA, 22_TERRA_2Ap7, 22_TERRA_2Ap13, and 22_TERRA_2Ap19 at 3 µM concentration. The buffer conditions were 10 mM potassium phosphate buffer (pH 7) and 10 mM KCl.

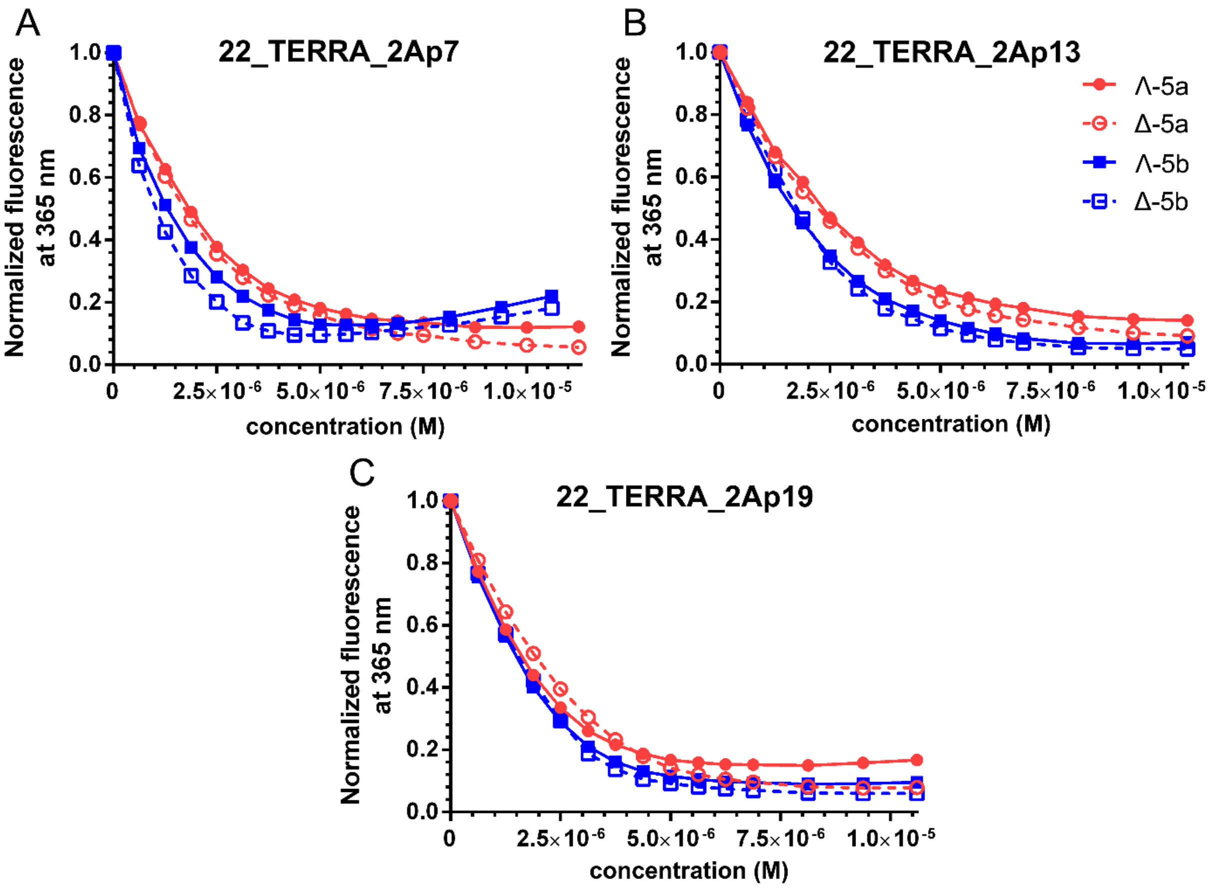

Figure S11. Fluorescence titrations of **22_TERRA_2Ap7** (A), **22_TERRA_2Ap13** (B), and **22_TERRA_2Ap19 (C)** at 2.5 μM concentration with Λ- and Δ-enantiomers of **5a** and **5b.** The buffer conditions were 10 mM potassium phosphate buffer (pH 7) and 10 mM KCl.

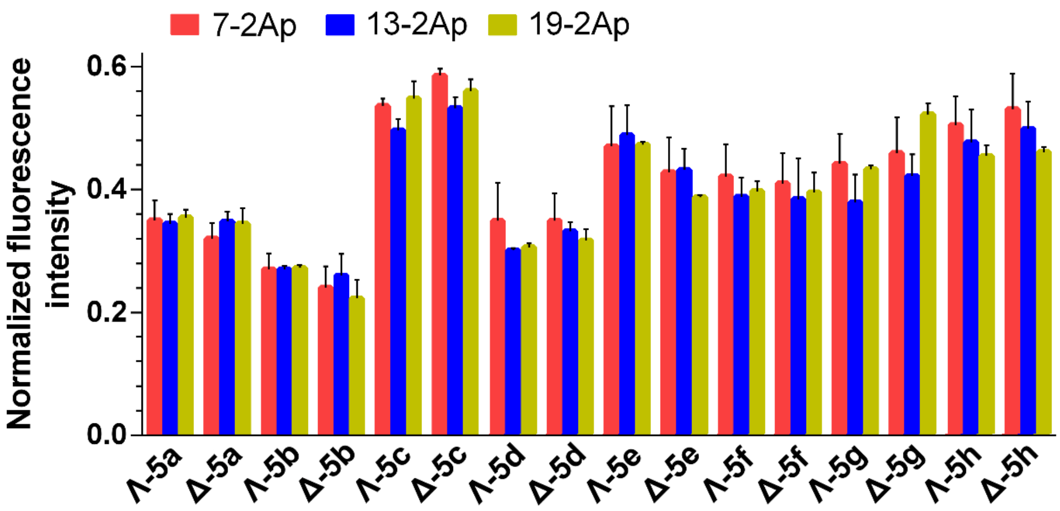

Figure S12. Influence of metallohelices (2.5 μM) on the fluorescence of **22_TERRA** (2.5 μM) labelled with 2-aminopurine (2Ap) at positions 7, 13, and 19 (see scheme in Figure 1B). The buffer conditions were 10 mM potassium phosphate (pH 7) and 10 mM KCl. Data shown are expressed as the mean of two independent experiments; error bars indicate the standard error of the mean.

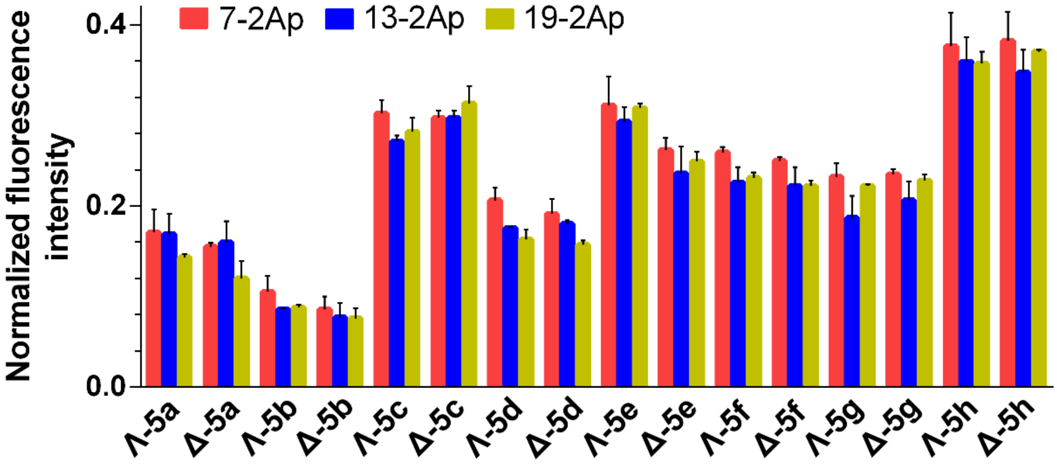

Figure S13. Influence of metallohelices (5 μM) on the fluorescence of **22_TERRA** (2.5 μM) labeled with 2-aminopurine (2Ap) at positions 7, 13, and 19 (see scheme in Figure 1B). The buffer conditions were 10 mM potassium phosphate (pH 7) and 30 mM KCl. Data shown are expressed as the mean of three independent experiments; error bars indicate the standard error of the mean.

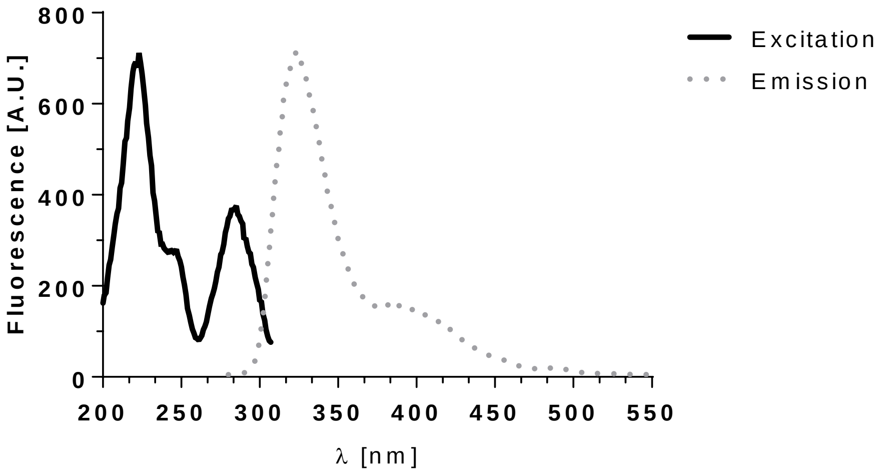

Figure 14. Excitation and emission spectra of **5h** in water.

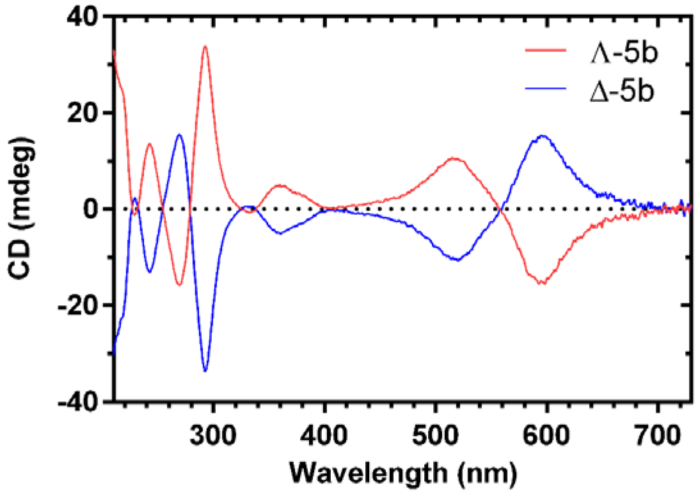

Figure S15. CD spectra of of **Λ-5b** and **Δ-5b** at the concentration of 10 μM in water.
